# Anti-CNTN1 IgG3 induces acute conduction block and motor deficits in a passive transfer rat model

**DOI:** 10.1186/s12974-019-1462-z

**Published:** 2019-04-05

**Authors:** Kathrin Doppler, Yasmin Schuster, Luise Appeltshauser, Lydia Biko, Carmen Villmann, Andreas Weishaupt, Christian Werner, Claudia Sommer

**Affiliations:** 1 Department of Neurology, University Hospital Würzburg, Josef-Schneider-Str. 11, 97080 Würzburg, Germany; 2 University Hospital Würzburg, Institute for Clinical Neurobiology, Versbacher Str. 5, 97078 Würzburg, Germany; 3 Department of Biotechnology and Biophysics, University of Würzburg, Am Hubland, 97074 Würzburg, Germany

**Keywords:** Paranodopathy, Anti-contactin-1, CIDP, Passive transfer, Autoantibody, Complement deposition

## Abstract

**Background:**

Autoantibodies against the paranodal protein contactin-1 have recently been described in patients with severe acute-onset autoimmune neuropathies and mainly belong to the IgG4 subclass that does not activate complement. IgG3 anti-contactin-1 autoantibodies are rare, but have been detected during the acute onset of disease in some cases. There is evidence that anti-contactin-1 prevents adhesive interaction, and chronic exposure to anti-contactin-1 IgG4 leads to structural changes at the nodes accompanied by neuropathic symptoms. However, the pathomechanism of acute onset of disease and the pathogenic role of IgG3 anti-contactin-1 is largely unknown.

**Methods:**

In the present study, we aimed to model acute autoantibody exposure by intraneural injection of IgG of patients with anti-contacin-1 autoantibodies to Lewis rats. Patient IgG obtained during acute onset of disease (IgG3 predominant) and IgG from the chronic phase of disease (IgG4 predominant) were studied in comparison.

**Results:**

Conduction blocks were measured in rats injected with the “acute” IgG more often than after injection of “chronic” IgG (83.3% versus 35%) and proved to be reversible within a week after injection. Impaired nerve conduction was accompanied by motor deficits in rats after injection of the “acute” IgG but only minor structural changes of the nodes. Paranodal complement deposition was detected after injection of the “acute IgG”. We did not detect any inflammatory infiltrates, arguing against an inflammatory cascade as cause of damage to the nerve. We also did not observe dispersion of paranodal proteins or sodium channels to the juxtaparanodes as seen in patients after chronic exposure to anti-contactin-1.

**Conclusions:**

Our data suggest that anti-contactin-1 IgG3 induces an acute conduction block that is most probably mediated by autoantibody binding and subsequent complement deposition and may account for acute onset of disease in these patients. This supports the notion of anti-contactin-1-associated neuropathy as a paranodopathy with the nodes of Ranvier as the site of pathogenesis.

**Electronic supplementary material:**

The online version of this article (10.1186/s12974-019-1462-z) contains supplementary material, which is available to authorized users.

## Background

Neuropathies with autoantibodies against paranodal proteins comprise a recently described subgroup of inflammatory neuropathies. So far, autoantibodies against the paranodal proteins contactin-1 (CNTN1), neurofascin-155 and contactin-associated protein (Caspr) have been identified [1–5]. Most patients with anti-CNTN1 autoantibodies show a distinct clinical phenotype of acute-onset severe sensorimotor peripheral neuropathy, in some associated with a disabling tremor and/or sensory ataxia [3, 4, 6]. Neuropathies with antibodies against paranodal proteins are often referred to as “paranodopathies” as the paranode is the site of immune attack [7, 8]. However, this term was originally introduced to classify anti-ganglioside autoantibody-associated acute motor axonal neuropathy with reversible conduction failure and referred to the pathophysiological concept of complement-mediated reversible conduction block [9, 10]. Autoantibodies against paranodal proteins mostly belong to the IgG4 subclass that does not activate complement, but IgG1, IgG2 and IgG3 autoantibodies have also been described either in combination with IgG4 or as the predominant subclass [3, 4]. Pathogenicity of IgG4, but not of IgG1, was recently demonstrated by intravenous passive transfer of anti-CNTN1 IgG4 to rats immunised with P2 peptide [2]. Further indicators of a pathogenic role of paranodal autoantibodies are the destruction of paranodal architecture detectable in patients with anti-CNTN1 associated neuropathy, the excellent therapeutic response to rituximab and the uniform clinical phenotype of the patients [4]. There is striking evidence that IgG4 autoantibodies play a pathogenic role during the course of disease and it was shown in vitro that anti-CNTN1 autoantibodies inhibit cell adhesion, which may account for anti-CNTN1 IgG4-induced structural damage of the paranodes [11]. However, the pathomechanism of acute onset of disease in these patients is still unclear, as structural changes induced by non-inflammatory IgG4 autoantibodies are unlikely to induce an acute phenotype. Accordingly, an immediate effect was absent after a single intraneural injection of anti-CNTN1 [2]. In a former study, we could detect anti-CNTN1 IgG3 in two patients with inflammatory neuropathy, both tested during the acute onset of disease [12] and anti-Caspr IgG3 in a patient with GBS phenotype [5]. We furthermore demonstrated that binding of anti-CNTN1 induces complement deposition and activation, related to the amount of IgG3 autoantibodies [13]. We therefore hypothesised that acute exposure of anti-CNTN1 IgG containing IgG3 may induce an acute neuropathy with reversible conduction failure, corresponding to the original concept of paranodopathy.

## Methods

### Patients, purification of IgG and determination of IgG subclasses

We used purified IgG of three patients with high titres of anti-CNTN1 autoantibodies. The clinical details and the procedure of anti-CNTN1 detection were recently reported [4]. IgG was purified from material of therapeutic plasma exchange by exchange chromatography as previously described [14]. Plasma exchange material of two patients with optic neuritis and without evidence of autoantibodies served as controls. The titres of IgG subclasses of the purified IgG were determined by enzyme-linked immunosorbent assay (ELISA) as previously described [4]. To determine the IgG subclass effectively binding to the paranodes, binding assays with teased fibres and subclass-specific FITC-conjugated secondary antibodies (anti-human IgG3: Merck, Darmstadt, Germany; anti-human IgG1, IgG2, IgG4: Abcam, Cambridge, UK) were performed as previously described [4]. Ethics approval was granted by the Ethics’ Committee of the University of Würzburg Medical Faculty.

### Animals, study design and intraneural injection

**Fig. 1:**
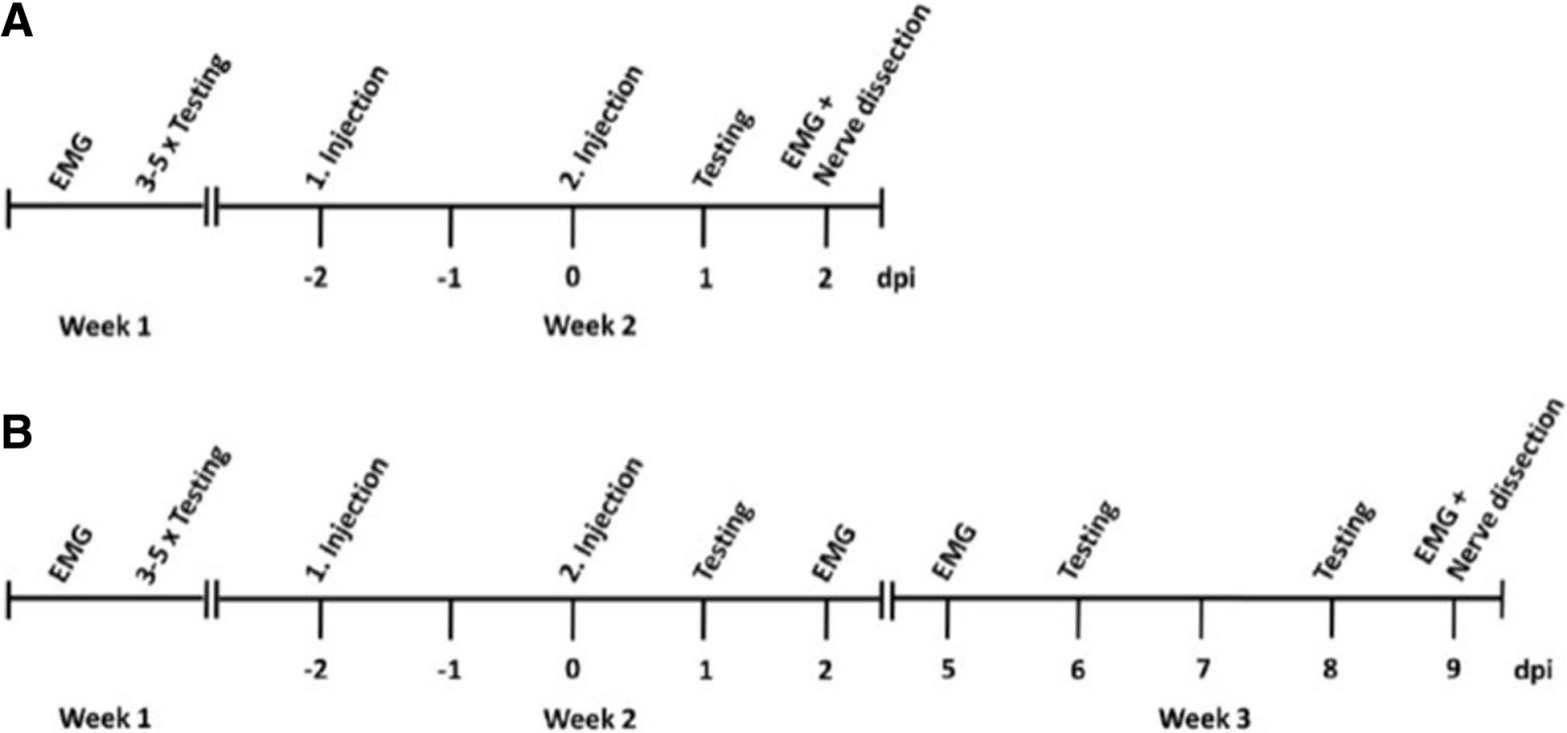
Schedule of the experiments. **a** Shows the standard course of experiments, in (**b**) an extended protocol for the study of recovery up to 9 days after injection is shown

Eight-week-old female Lewis rats were obtained from Charles River (Sulzfeld, Germany). Animals were kept in cages with free access to water and food. All experiments were approved by the Bavarian State authorities (Regierung von Unterfranken). Intraneural injection of 10 μl of patient IgG (100 mg/ml) was performed under anaesthesia with isoflurane by dissecting the right sciatic nerve at the sciatic notch, following injection with a 27G Hamilton syringe. The timeline of the experiments is demonstrated in Fig. 1. All animals were injected at two time-points. The week before the injection, nerve conduction studies (NCS) and 4 days of behavioural testing were performed. Behavioural testing was repeated the day after the second injection. NCS were performed 2 days after the second injection, and tissue processing was done immediately after the NCS. To study potential recovery of the animals from autoantibody injection, an extended experiment was conducted in seven animals, repeating behavioural testing and NCS twice in the week after injection (Fig. 1b). All injections, behavioural tests and NCS were conducted under blinded conditions. Rats were randomised to the patient/control groups and to the extended study by a person who was not involved in the experiments. Blinded aliquots of patient/control IgG were prepared by this person and the random list remained sealed until the end of data acquisition.

### Behavioural testing

For motor testing, the rats were put on an accelerating RotaRod (TSE Systems, Bad Homburg, Germany) and the mean latency time to fall of five trials was calculated for each animal. For statistical comparison, the ratio of fall latencies pre- and post-injection for each animals was calculated and compared between patients and controls. Gait analysis was performed using the Catwalk XT (Noldus, Emmerich am Rhein, Germany), a transparent glass runway with a camera underneath that detects the footprints. The following parameters were calculated: standing time, print area and maximum intensity. We recorded three runs per animal and the mean value of each parameter was calculated and the ratio of pre- and post-injection values was taken. Sensory function was assessed by von-Frey filaments. The left and right hindpaws were tested six times each and were analysed using Dixon’s staircase system [15].

### Nerve conduction studies

All NCS were performed under intraperitoneal anaesthesia with ketamine/xylazine that does not reduce F wave persistence [16]. The left and right sciatic/tibial nerve of each animal was measured using Neurosoft-Evidence 3102 electromyograph (Schreiber and Tholen Medizintechnik GmbH, Stade, Germany) and the appropriate software. The skin temperature was maintained at 34–36 °C using a warming lamp. For motor NCS, the recording electrodes were placed into the sole of the hindpaw with the active electrode between the third and fourth toe and the inactive electrode lateral to the first toe. The distal stimulation electrodes were placed above the ankle, the proximal stimulation electrodes at the sciatic notch. For motor neurographies, a supramaximal stimulus was applied at the distal and proximal stimulation site and the distance between both sites was used for the calculation of the nerve conduction velocity (NCV). F waves and H-reflexes were obtained by ten serial supramaximal stimuli with frequencies of 0.3, 1, and 10 Hz at the distal stimulation site. The minimum F wave latencies and the persistence of F waves was obtained for each frequency, the persistence of H-reflexes was only obtained at 0.3 Hz because no H-reflexes were recordable at higher frequencies. For NCS of mixed afferents, the stimulation electrodes were placed above the ankle and the recording electrodes at the sciatic notch. NCV was calculated from the distance between stimulation and recording electrodes. Electromyography was performed in the small foot muscles and the gastrocnemius muscles by needle electrodes.

### Histological and immunohistochemical analysis

After the last NCS, the rats were sacrificed and the sciatic nerve was rapidly removed and was cut into three pieces: the proximal one was mounted in Tissue-Tec and was deeply frozen in liquid nitrogen for immunohistochemistry, the middle one was immediately prefixed in 4% paraformaldehyde for 10 min for teased fibre preparations and the distal one was fixed in glutaraldehyde for semi-thin sections. All three pieces were supposed to be reached by the injection as they were only millimetres apart from the injections site. For immunohistochemical analysis of inflammation, 20-μm cross sections were cut, fixed with acetone for 10 min, blocked with 10% bovine serum albumin (BSA)/phosphate-buffered saline (PBS) for 30 min and were incubated with the following primary antibodies diluted in PBS with 0.3% TritonX overnight: anti-CD3 (rabbit, 1:500, Abcam), anti-C5b9 (mouse, 1:1000, Dako/Agilent Technologies, Santa Clara, California, USA, mouse, 1:500, Santa Cruz, Dallas, Texas, USA), anti-C1q (mouse, 1:50, Abcam), anti-CD68 (mouse, 1:1000, Dako) and Cy3-conjugated anti-rabbit/anti-mouse secondary antibodies (Dianova, Hamburg, Germany). For semi-thin sections, tissue was fixed with 4% paraformaldehyde/glutaraldehyde for at least 24 h, was post-fixed with 2% osmiumtetraoxide, dehydrated by acetone and was embedded in 3,4-epoxycyclohexylmethyl. Semi-thin sections were cut using a microtome (Leica EM UC7, Leica, Wetzlar, Germany) and were stained with azure II and methylene-blue.

For the analysis of nodal architecture, immunofluorescence was performed as previously described [5]. Shortly, slides were fixed with acetone, incubated with primary antibodies (anti-Caspr, Abcam, 1:100; anti-pan-neurofascin, Abcam, 1:400, anti-pan-sodium-channel, Sigma Aldrich, St. Louis, Missouri, USA, 1:100) overnight at 4 °C and with appropriate secondary antibodies (Alexa-Fluor488-conjugated anti-rabbit or anti-mouse IgG, 1:200, Dianova) for 2 h at room temperature.

Immunofluorescence was analysed using a fluorescence microscope (Axio Imager.M2, Zeiss, Oberkochen, Germany); semi-thin sections were analysed by light microscopy (Olympus BH2, Olympus, Shinjuku, Japan).

### Structured illumination and confocal microscopy

Immunostained teased fibres were recorded on a commercially available SIM Zeiss ELYRA S.1 microscope using a 63×/1.40 oil immersion objective. Laser illumination at 568 nm was used for excitation of Cy3. Images were recorded with structured illumination applying five rotational and five phase variations. The final superresolved image was reconstructed in ZEN software (Zeiss) and rendered in Imaris (Bitplane, Belfast, UK).

Confocal microscopy images of anti-Caspr staining of teased fibres were recorded on a commercial LSM700 (Zeiss) setup using a 63×/1.4 oil immersion objective. Morphometry by confocal microscopy was restricted to a subset of four to six animals per patient. At least eight nodes per animal were measured. The same number of matched controls was measured in parallel with each patient animal. Z-stacks were acquired with a pixel size of 91 nm and a z-step size of 300 nm. Cy3 signal was recorded using a DPSS laser line (555 nm) and suitable filter settings. Z-stacks were loaded in Imaris for further analysis. After applying intensity based thresholding (constant thresholding values for both control and patient), area, volume and intensities of singular Caspr patches were exported using Excel (Microsoft). Length measurements were performed by hand using the section and measurement tool in Imaris. Representative images were processed in ImageJ (FIJI distribution [17]). Processing included linear correction using brightness and contrast only.

### Statistical analysis

Statistical analysis was performed using SPSS 23.0 (IBM, Armonk, New York, USA). Normal distribution of data was tested by Shapiro-Wilk test. For normally distributed data, a *t* test was performed; for non-normally distributed data, we used Mann-Whitney *U* test. Pairwise comparisons were done by related samples Wilcoxon signed rank test. Statistical comparisons for microscopy data were performed in Origin (OriginLab, Northampton, Massachusetts, USA). Mann-Whitney *U* test was applied to make single comparisons between control and patient group.

## Results

### Patients and antibody titres

**Fig. 2:**
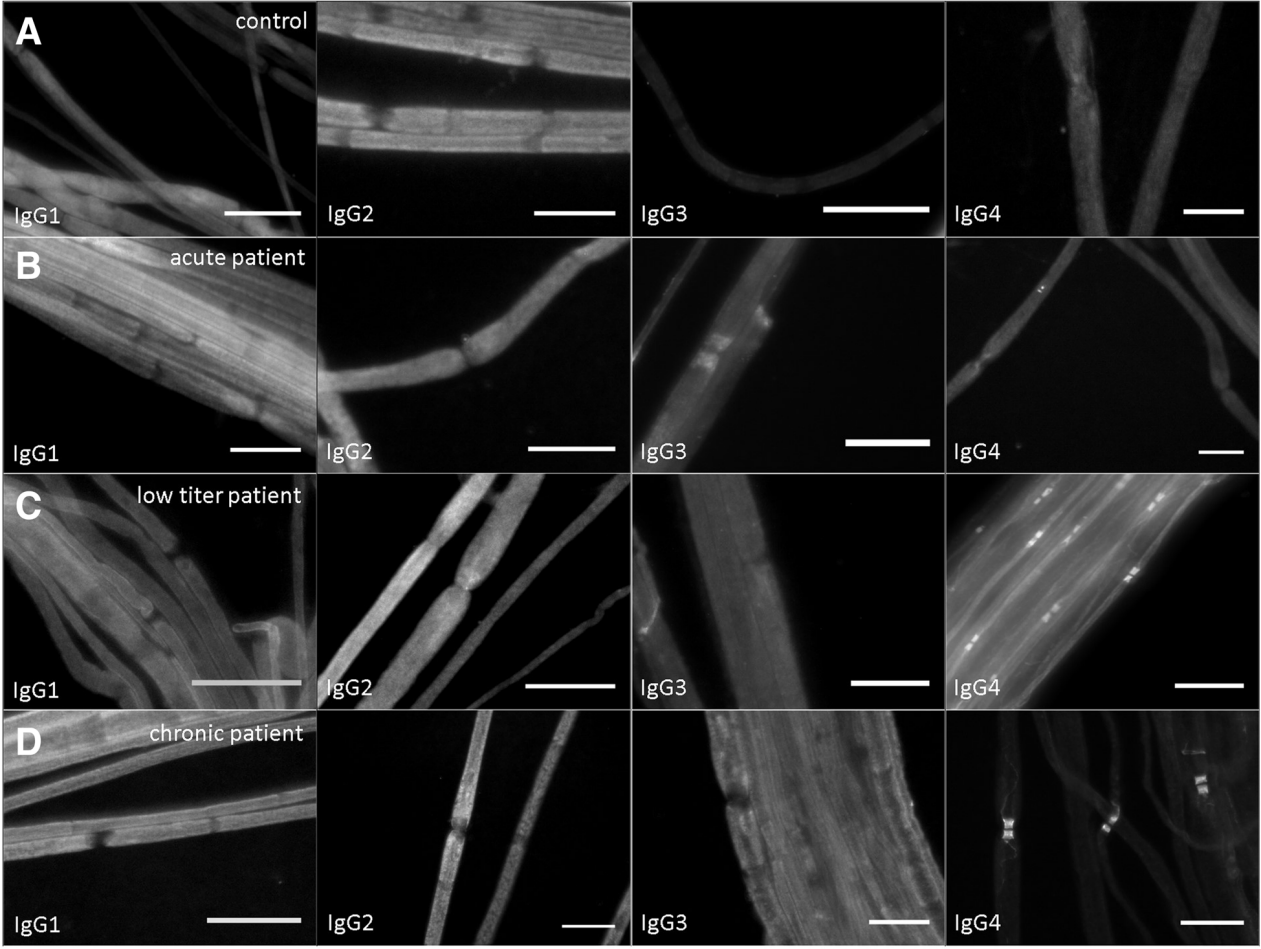
Binding assay with patients’ sera on murine teased fibres using subclass-specific secondary antibodie**s.** No binding to paranodes is visible after incubation with a control serum (**a**). The acute patient shows paranodal deposition of IgG3 and weakly of IgG4 (**b**). In the low titre and chronic patient, IgG4 can be identified as the binding subclass (**c**, **d**). Bar = 20 μm

Purified IgG of three patients with anti-CNTN1-associated neuropathy was used for the experiments. Anti-CNTN1 autoantibodies of these three patients were identified in a former study by ELISA and all three patients showed distinct binding to the paranodes of murine teased fibres and to CNTN1-transfected human embryonic kidney 293 cells [4]. The ELISA antibody titres of the purified IgG of the individual patients are shown in Table 1. In one patient whose IgG was obtained during the acute onset of disease (further referred to as “acute patient”), IgG3 was the predominant subclass (1:7500) with only low titres of IgG1 (1:1000) and IgG4 (1:500). In the second patient (also recruited during the chronic phase of disease), low titres of all four subclasses were detectable with predominance of IgG3 (1:1000) and IgG4 (1:1000) (further referred to as “low titre patient”). The third patient whose serum was also obtained during the chronic phase of disease showed high titres of IgG4 (1:15,000), a moderate titre of IgG3 (1:5000) and low titres of IgG1 (1:500) and IgG2 (1:1000) (further referred to as “chronic patient”). Binding assays on murine teased fibres using subclass-specific secondary antibodies revealed IgG3 and IgG4 as the binding subclasses in the acute patient and only IgG4 in the other two patients (Fig. 2).

**Table 1:** Summary of antibody titres and subclasses, electrophysiological and morphological data of rats injected with IgG of different patients. Significant differences (between patients and controls) are marked with*

Rats injected with IgG of		Acute patient (*n* = 12)	Low titre patient (*n* = 15)	Chronic patient (*n* = 20)	Control animals (*n* = 36)
Titre of IgG subclasses (predominant subclass in bold type)	IgG1	1:1000	1:100	1:500	Negative
	IgG2	negative	1:100	1:1000	
	IgG3	**1:7500**	**1:1000**	1:5000	
	IgG4	1:500	**1:1000**	**1:15,000**	
Binding subclass	In vitro	IgG3, (IgG4)	IgG4	IgG4	None
	In vivo	IgG3	No binding detectable	IgG4	
Conduction block		83.3%*	0%	35%	13.2%
Loss of F waves		66.7%*	0%	40%*	0%
Median CMAP ratio (distal/proximal) pre-/post-injection (mV)		1.14 (1.03–1.34)	1.18 (1.04–1.24)	1.11 (0.89–1.38)	1.12 (0.88–2.49)
		3.32 (1.04–20.25)*	1.18 (0.92–1.71)	1.35 (1.07–13.35)	1.23 (1.08–5.12)
Median SNAP pre-/post-injection (μV)		20.0 (6.9–29.9)	17.4 (10.0–59.9)	18.0 (0–26.1)	17.8 (2.9–106)
		8.8 (0–18.7)*	13.2 (0.3–63.0)	13.7 (1.3–30.9)	15.7 (3.6–39.2)
Median fall latency (s)		205 (111–336)	230 (64–396)	231 (100–500)	223 (71–401)
		155 (54–222)*	186 (76–297)	185 (84–389)	180 (67–343)
Median standing time right hind paw (s)		0.23 (0.17–0.28)	0.24 (0.19–0.37)	0.22 (0.17–0.52)	0.24 (0.15–0.46)
		0.18 (0.08–0.25)*	0.24 (0.18–0.45)	0.22 (0.11–0.44)	0.24 (0.15–0.43)
Median print area right hind paw (cm^2^)		1.39 (0.54–2.18)	1.89 (1.04–2.74)	1.18 (0.45–1.73)	1.30 (0.64–2.35)
		0.88 (0.09–2.21)*	2.07 (1.30–2.98)	1.04 (0.32–1.51)	1.61 (0.74–2.91)
Median maximum intensity right hind paw (AU)		217 (182–229)	222 (207–232)	212 (180–219)	210 (173–232)
		177 (83–234)*	220 (206–234)	202 (94–221)	215 (165–235)

### IgG3 and IgG4 autoantibodies access paranodes after intraneural injection

**Fig. 3:**
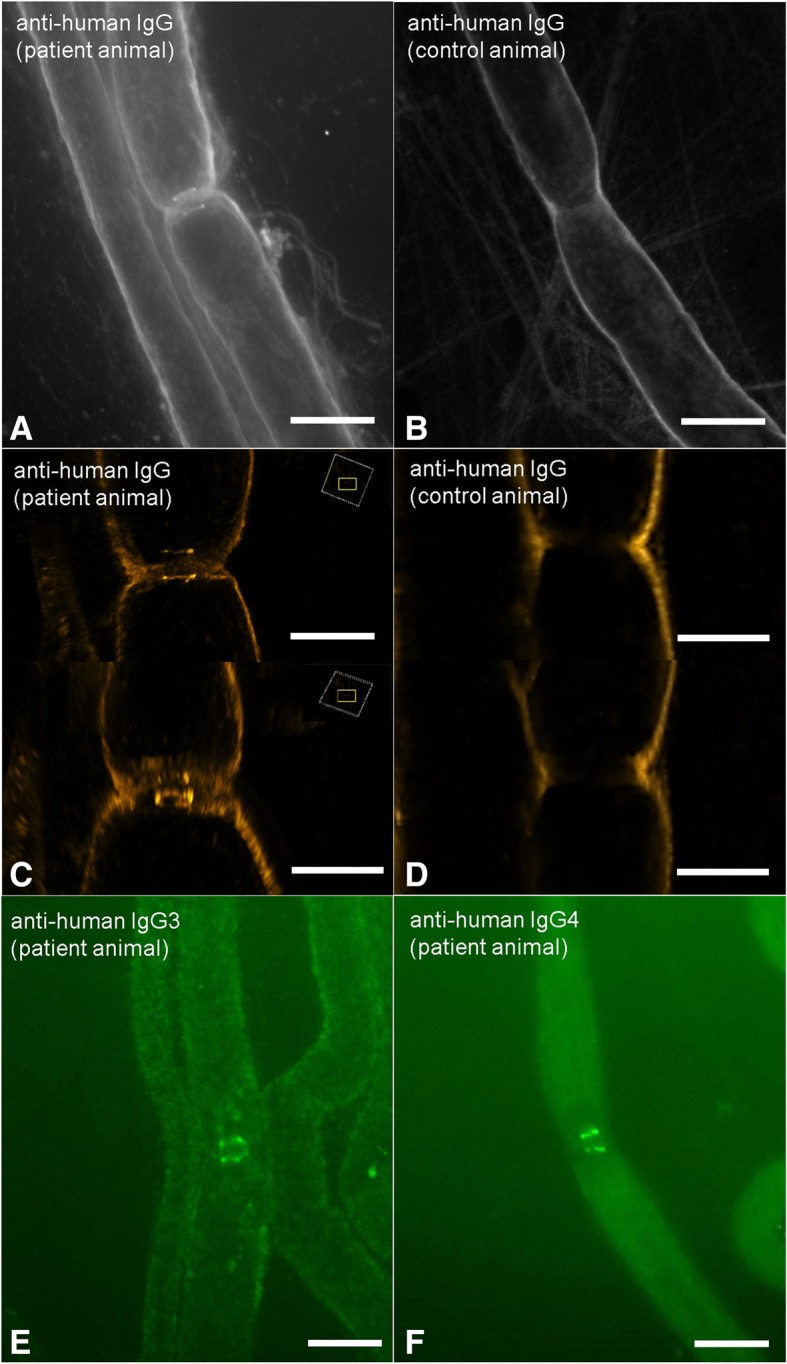
Immunofluorescence staining of sciatic nerves of rats with Cy3-conjugated anti-human IgG (**a**, **b**), FITC-conjugated anti-human IgG3 (**c**) and IgG4 (**d**). Confocal fluorescence microscopy detects a small band of autoantibody binding at the paranodes of a patient animal (**a**), not in a control animal (**b**). Structural illumination microscopy reveals a tube-like arrangement of autoantibodies around the axon in the patient animal (**c**), not in the control animal (**d**). Subclass specific antibodies uncover IgG3 as the binding subclass in the acute patient (**e**) and IgG4 in the chronic patient (**f**). Bars in (**a**, **b**, **e**, **f**) = 10 μm, in (**c**) and (**d**) 5 μm, rectangles in (**c**) indicate the orientation

To confirm the binding of patient IgG to the paranodes after intraneural injection, teased fibres of the sciatic nerves of all rats were incubated with Cy3-conjugated anti-human IgG. Positive binding 2 days after injection was detectable in 33.3% of animals injected with IgG of the acute patient, in 20% of the animals of the chronic patient and in none of the animals injected with IgG of the low titre patient or the control patients. Binding did not include the whole paranodal region but was restricted to a small band adjoining the nodes, presumably those parts of the paranodes that are easily accessible by autoantibodies in vivo (Fig. 3a). Structured illumination microscopy revealed a tube-like arrangement of autoantibody binding, spanning the circumference of the nerve fibres (Fig. 3b). To further specify the subclass of IgG that binds to the paranodes, teased fibres of rats injected with IgG of the acute and chronic patient were incubated with anti-human IgG3 and IgG4. In the acute patient, binding of IgG3 but not IgG4 to the paranodes could be detected; in the chronic patient, IgG4 but not IgG3 was the binding subclass, confirming the predominant IgG subclass as the binding subclass and demonstrating that IgG3 as well as IgG4 autoantibodies are able to access the paranodes in vivo (Fig. 3c, d).

### Conduction blocks in animals injected with patient IgG

**Fig. 4:**
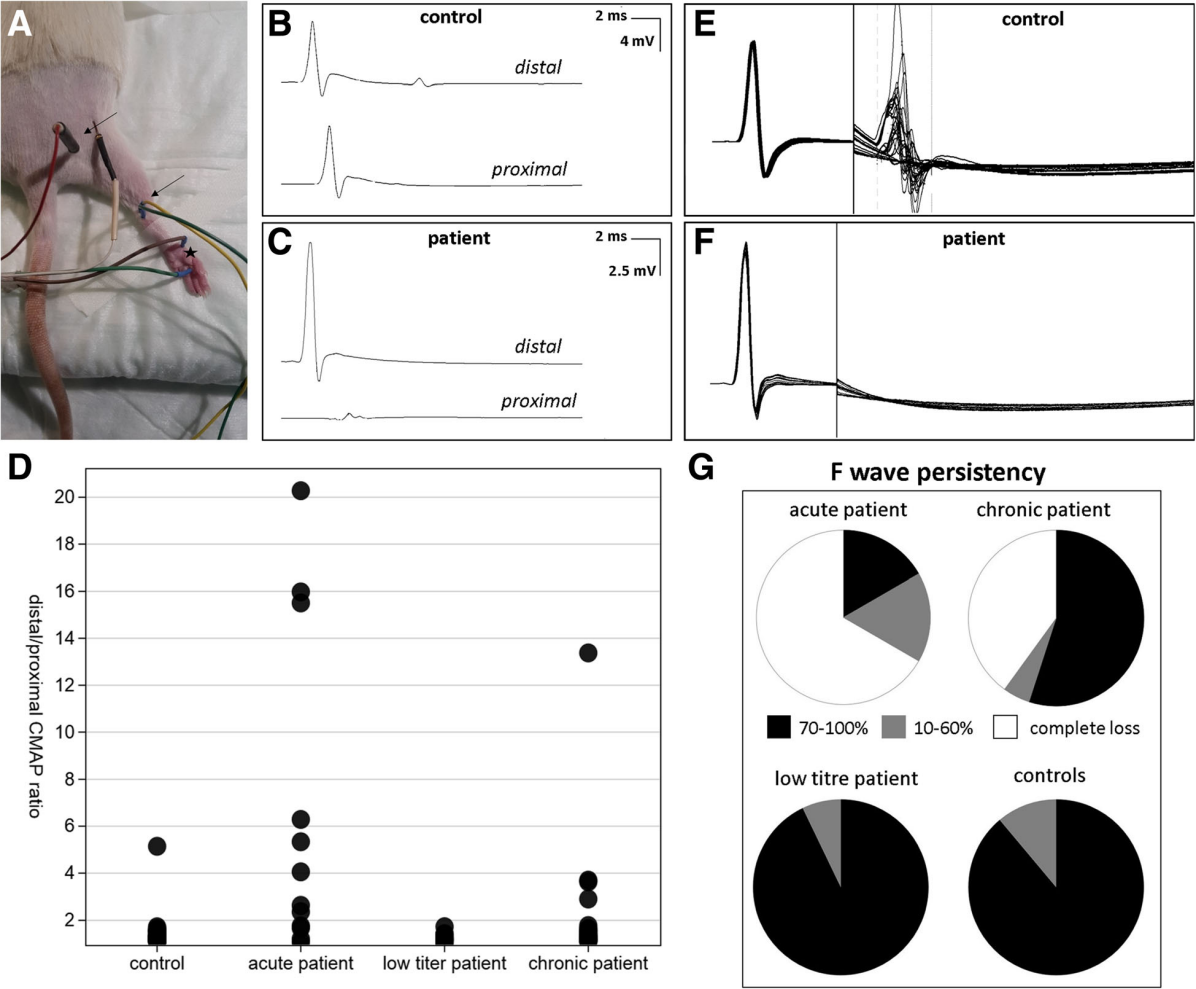
Summary of results of nerve conduction studies (NCS). Sciatic motor NCS were performed by stimulating above the ankle (distal site) or at the sciatic notch (proximal site) and recording from the sole (**a**). Stimulation sites are marked by arrows, the recording site by the asterisk. Almost equal CMAP amplitudes were measured in controls (**b**), conduction blocks defined as a ratio of the distal to proximal CMAP of > 1.5 were found in patient animals (**c**). The ratio of distal to proximal CMAP was increased in animals injected with IgG of the acute patients compared to controls (**d**). F waves following the CMAP were measured in controls (**e**), whereas loss of F waves was found in patient animals (**f**). F wave persistence was mostly normal (70–100%, black) in animals injected with IgG of the low titre patient and controls, whereas complete loss of F waves (white) was found in the majority of animals injected with IgG of the acute patient and many animals injected with IgG of the chronic patient (**g**)

Conduction blocks defined as a ratio of the distal and proximal compound muscle action potential (CMAP) of more than 1.5 were measured in 83.3% of rats injected with IgG of the acute patient and in 35% of rats injected with IgG of the chronic patient, but also in 13.2% of control animals as a side-effect of intraneural injection. No conduction blocks were detectable in the rats injected with IgG of the low titre patient. Loss of F waves in combination with a normal distal CMAP, a further indicator of proximal conduction block, was found in 66.7% of rats injected with IgG of the acute patient and in 40% with IgG of the chronic patient, but not in any rat injected with IgG of the low titre patient or any control animal. The combination of conduction block and loss of F waves was found in 66.7% of animals of the acute patient and in 25% of animals of the chronic patient, not in any control (Fig. 4). Distal motor latency did not differ between patient animals and controls. We did not detect any conduction block or loss of F waves prior to injection.

Accordingly, the ratio of the right distal and proximal CMAP after injection was increased in animals injected with IgG of the acute patient compared to control animals (*p* = 0.0001) and tended to be higher in animals injected with IgG of the chronic patient in comparison with controls (*p* = 0.092) but did not differ in animals of the low titre patient. The persistence of F waves on the right side after injection was reduced in animals of the acute and chronic patient (acute patient: 0.3 Hz *p* = 0.0001, 1 Hz *p* = 0.0001, 10 Hz *p* = 0.001; chronic patient: 0.3 Hz *p* = 0.015, 1 Hz *p* = 0.024, 10 Hz p = 0.0001), not in animals of the low titre patient. No differences of these parameters between groups were recorded before injection or on the left side. In 41.7% of rats injected with IgG of the acute patient, but not in any control or other patient animal, no sensory nerve action potential (SNAP) was recordable after injection, presumably due to a sensory conduction block. All these animals also had a motor conduction block. We did not detect any spontaneous activity by EMG.

### Motor deficits after injection of patient IgG

**Fig. 5:**
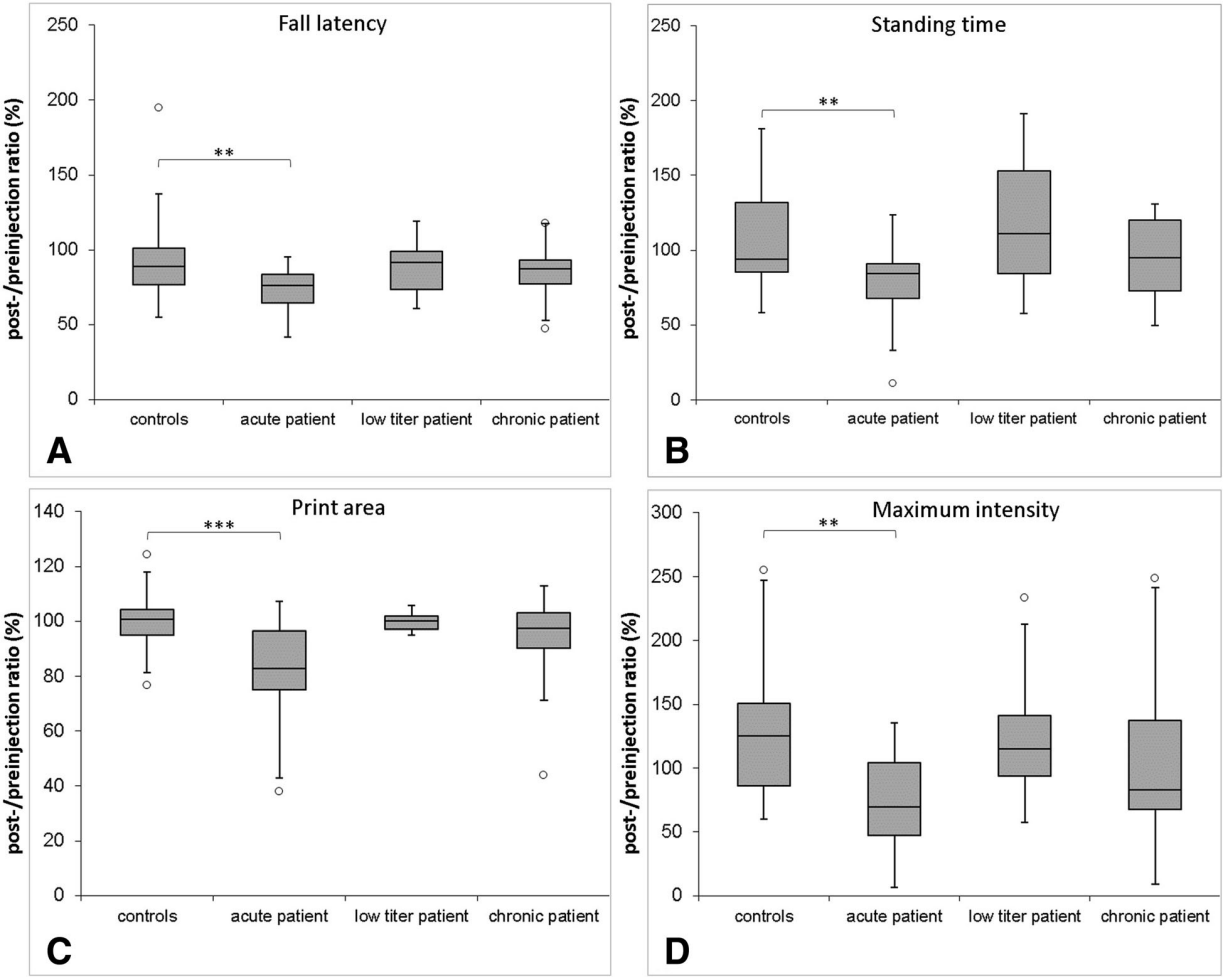
Box plots of parameters of motor testing**.** The *y*-axis gives the values after injection in relation to pre-testing values (in %). Fall latency measured by rotarod is decreased in animals injected with IgG of the acute patient (**a**). Catwalk gait analysis reveals decreased standing time, print area and maximum intensity of the right hind paw in animals injected with IgG compared to controls (**b**–**d**). The boxplots show medians and quartiles; circles represent outliers. (*0.1 > *p* > 0.01, **0.01 > *p* > 0.001, ***0.001 > *p*)

Rotarod testing indicated motor deficits in rats injected with IgG of the acute patient: here, the latency to fall after injection in relation to latency pre-injection was decreased compared to controls (*p* = 0.0058). No differences were detectable between the fall latencies of rats injected with IgG of the other patients and controls (Fig. 5a).

Gait analysis with the Catwalk system showed that rats injected with IgG of the acute patient had a decreased relative standing time (*p* = 0.0040, Fig. 5b), a reduced relative foot print area (*p* = 0.0003, Fig. 5c) and a reduced relative maximum intensity (*p* = 0.0013, Fig. 5d) on the right hind paw compared to control rats, reflecting limping of the right hind paw post-injection. Rats injected with IgG of the chronic and low titre patient did not show a relative reduction of any of these parameters after injection.

No differences in the von Frey testing were observed in rats injected with patient IgG and controls (data not shown).

### Recovery of nerve conduction and motor deficits over time

**Fig. 6:**
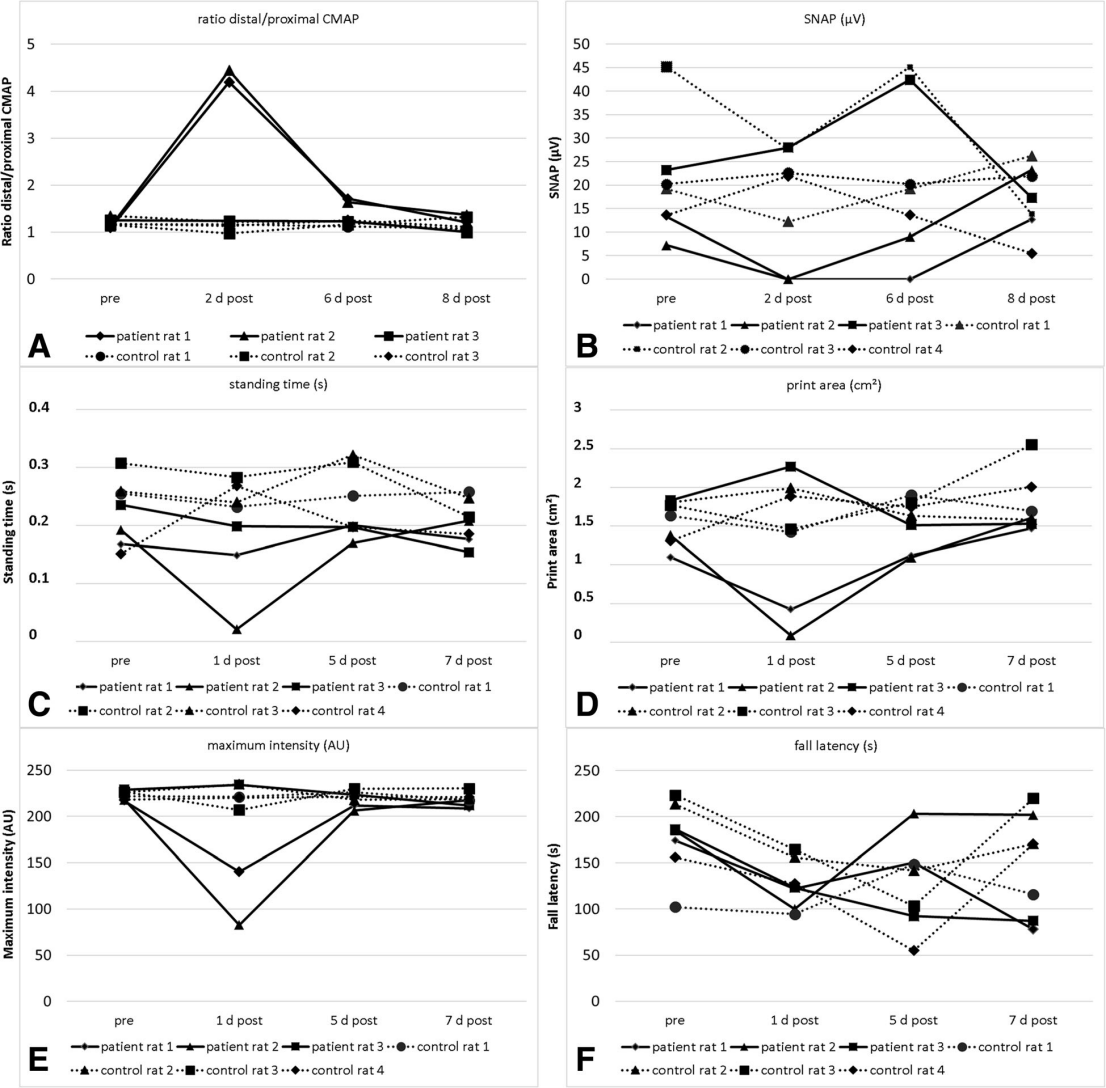
Change of NCS and motor testing parameters over time of three rats injected with IgG of the acute patient in comparison to four controls**.** Increase of distal/proximal CMAP ratio (**a**) and decrease of SNAP (**b**), standing time (**c**), print area (**d**), maximum intensity (**e**) and fall latency (**f**) of the right hind paw are detectable in two rats 1/2 days after injection and are almost normal four days later. Rat 3 did not show any effect (probably due to technical problems)

To study the time course of nerve conduction and motor deficits after injection, three animals injected with IgG of the acute patient and four animals injected with IgG of a control were tested at 1, 5 and 7 days after injection and had nerve conduction studies at 2, 6 and 8 days after injection (Fig. 1b). A conduction block, loss of F waves and motor deficits were detectable in two of the three rats injected with patient IgG 2 and 1 day after injection respectively. CMAP ratios and motor function were almost normal 6 and 5 days after injection respectively and were entirely normal at 8 and 7 days after injection (Fig. 6).

### Alterations of paranodal proteins after injection

**Fig. 7:**
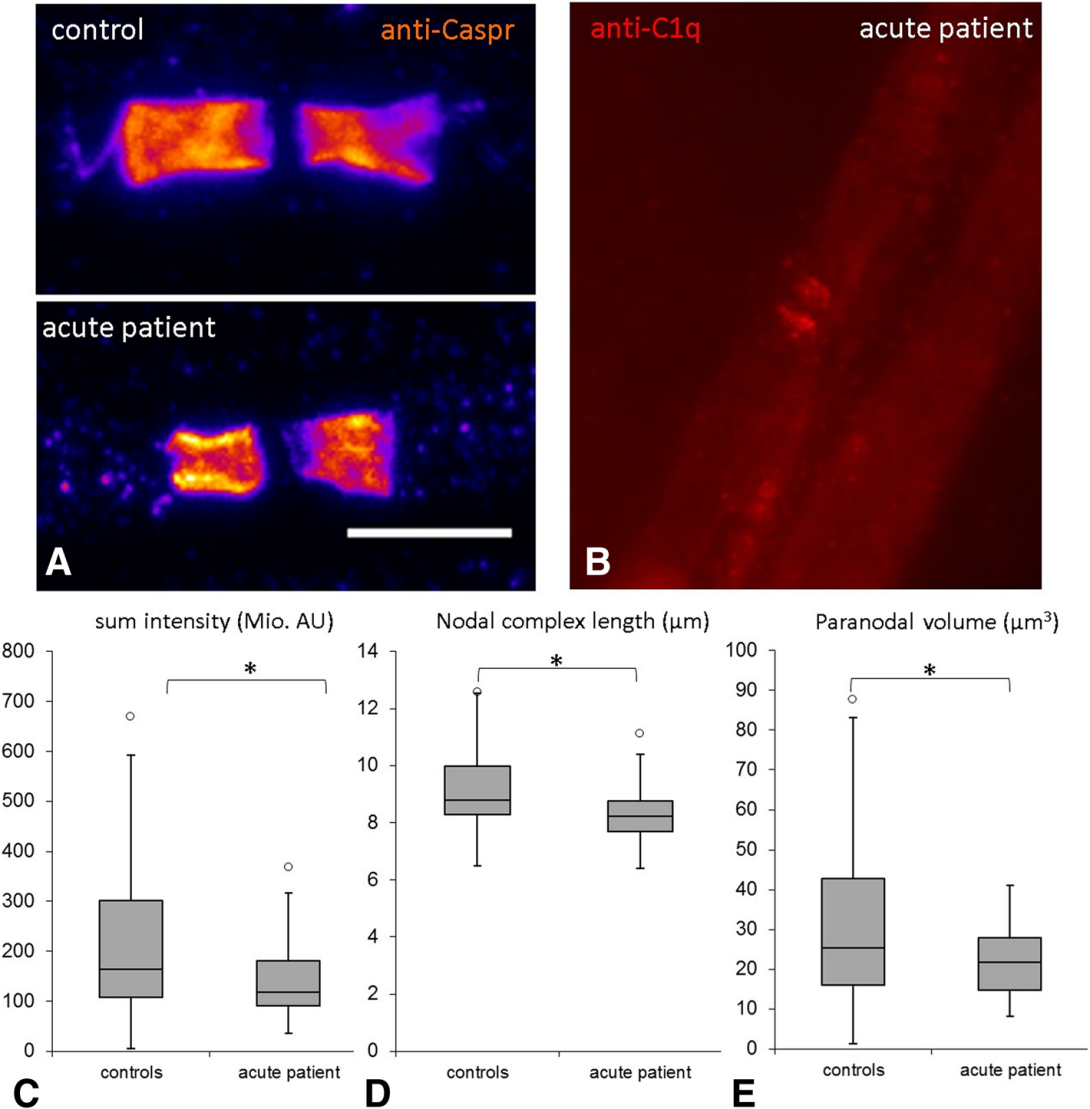
Confocal microscopy image of paranodes and box plots illustrating morphometric data and complement deposition of the acute patient**.** Paranodal immunofluorescence staining with anti-Caspr is reduced at paranodes of rats injected with IgG of the acute patient (**a**, lower image) compared to paranodes of a control rat (**a**, upper image). Scale bar = 10 μm. Complement deposition at the paranodes can be detected by immunofluorescence staining with anti-C1q (**b**). Morphometric analysis revealed a reduction of sum intensity (**c**), length of the nodal complex (**d**) and paranodal volume (**e**) in rats injected with IgG of the acute patient compared to control rats. (*0.1 > *p* > 0.01, **0.01 > *p* > 0.001, ***0.001 > *p*)

The distribution of the paranodal protein Caspr, the nodal and paranodal protein neurofascin and nodal sodium channels was studied by conventional immunofluorescence. We did not detect any abnormalities of sodium channel staining, neither a dispersion of sodium channels to the paranodes nor a weakened staining. We also did not detect any dispersion of Caspr or neurofascin to the juxtaparanodes or any elongated nodal gaps. As the short time course of anti-CNTN1 application may lead to minor alterations of signal distribution, we applied quantitative confocal microscopy on Caspr staining of teased nerve fibres of injected rats. Recordings revealed a modest reduction in signal volume, a mild reduction of length of the nodal complex and total signal intensity in rats treated with IgG of the acute patient, not in animals of the other patients or controls. The length of the nodal gap remained unchanged (Fig. 7, Additional file 1: video 1 and Additional file 2: video 2).

### Inflammatory cells and complement deposition

Paranodal deposition of complement component C1q was detectable at some nodes of animals injected with IgG of the acute patient that also showed paranodal IgG deposition but not in any animal injected with IgG of the chronic patient (Fig. 7b). A moderate increase of macrophages, T cells and C5b9 deposition was detectable in nerves on the right side compared to the left and may be induced by an inflammatory response to the injection (data not shown). We did not detect any differences between animals treated with patient IgG or control IgG, arguing against an inflammation-induced damage to the nerve fibres.

Semi-thin sections revealed acute Wallerian degeneration of some nerve fibres, probably due to mechanical damage. There were no differences between control and patient IgG injected animals (data not shown).

## Discussion

In this study, we detected conduction blocks and motor deficits in rats intraneurally injected with IgG of patients with anti-CNTN1-associated neuropathy. By choosing a short-term and focal application of IgG, we were able to demonstrate reversible conduction blocks accompanied by complement deposition, motor dysfunction, fitting with the original concept of paranodopathy as an autoantibody-mediated reversible dysfunction of the node of Ranvier. Even though the concept of paranodopathy has been expanded to the spectrum of IgG4-autoantibody-mediated neuropathies in the last years, our data give evidence that at least during the acute onset of disease reversible conduction block may be induced.

When comparing the effect of IgG of the acute phase of disease (predominantly IgG3) with IgG of the chronic phase (predominantly IgG4), we found a more severe effect of the “acute IgG”. This observation supports the idea of acute conduction block induced by autoantibody binding that has been extensively studied in anti-ganglioside-associated neuropathies [10, 18]. As described in studies on anti-ganglioside antibodies, deposition of C1q was detectable at the paranodes of rats injected with IgG of the acute patient. No C1q was detectable at paranodes of rats injected with IgG of the chronic patient, although complement binding was demonstrated in vitro in another study using sera of the very same patient [13]. This is most probably explained by the observation of IgG4 as the binding subclass and lack of detection of paranodal IgG3 binding in this patient. However, complement deposition was not detectable at all nodes. This might be a failure of detection due to temporary or only mild complement deposition on just those nodes that were reached by diffusion of IgG. In contrast to gangliosides that are found at the nodes and paranodes, CNTN1 is restricted to the paranodes that are not as easily accessible. This is most probably also the reason why we could only detect autoantibody binding in a small band not comprising the whole paranodal region. This observation is well in line with a previous study that demonstrated that binding to the complete paranodal region can only be seen after 3 days of antibody exposure [2]. The lifespan of anti-CNTN1 after intraneural injection was shown to be small with a significant decrease after 3 days [2]. So complement deposition may not be detectable anymore at all nodes at the day of tissue harvesting, impeded by autoantibody binding only to a small area of the paranode. Alternatively, autoantibodies may induce temporary axo-glial detachment, leading to leakage of driving currents that may contribute to conduction failure. The lack of spontaneous muscle activity in our animals supports the idea of a short-term pathogenic effect by conduction block without any axonal damage. Predominance of motor conduction deficits and motor dysfunction reflects the clinical phenotype of the patients who all suffered from motor more than sensory peripheral neuropathy [4].

It has been shown in vitro that anti-CNTN1 autoantibody binding of patients with predominance of IgG4 prevents adhesive interaction between CNTN1 and its binding partners Caspr and neurofascin-155 [11]. Structural changes of the paranodal region are therefore supposed to be involved in the pathogenicity of anti-CNTN1-associated neuropathy. Several studies demonstrated disruption of the paranodes in patients with anti-CNTN1-associated neuropathy [4, 19]. In contrast to studies of human nodal architecture, that demonstrated elongated nodes, a dispersion of paranodal proteins and sodium channels and a weakened staining of Caspr and neurofascin [4], we only detected a slightly reduced volume and sum intensity of paranodal proteins accompanied with minor differences of length of the nodal complex, no elongated nodes or dispersion of proteins. This is most probably explained by the short time course. Structural changes, as shown to be induced by anti-CNTN1 IgG after incubation for 36 h in vitro [11], may need a longer exposure to autoantibodies in vivo and it was shown that anti-CNTN1 autoantibodies have a short lifespan in vivo [2]. Reduced sum intensity and volume of Caspr staining, the paranodal binding partner of CNTN1, may be the result of cross-linking IgG3 autoantibodies that induce internalisation. However, the exact pathomechanisms need to be explored in future studies.

Our data provide new insights into the pathogenicity of acute autoantibody exposure in anti-CNTN1-associated neuropathy and extend recent findings that were obtained by a study from Manso et al.: by modelling chronic autoantibody exposure using intravenous application, they demonstrated a pathogenic effect of IgG4 anti-CNTN1, leading to exacerbation of neuropathy in animals immunised with P2 protein [2]. They did not succeed in inducing conduction block after intraneural injection of IgG4 or IgG1 anti-CNTN1 [2]. This can be explained by differences of IgG subclasses: the strongest effect in our study was measured in the acute patient with a high titre of IgG3 anti-CNTN1 and the chronic patient also had a relevant titre of IgG3.

Data of the study by Manso et al. and ours taken together support the idea that binding of IgG3 anti-CNTN1 as a complement-activating IgG subclass leads to an acute conduction block that is detectable after short-term injection and that IgG4 pathogenicity requires chronic autoantibody exposure. Acute onset of disease may display the clinical correlate of IgG3-mediated pathogenicity, whereas the chronic course of disease could be explained by effect of IgG4. Accordingly, the serum of the patient with predominance of IgG3 was obtained during the acute beginning of the disease, whereas the sera of the two other patients with IgG4 were obtained after several years of neuropathic symptoms as described in a previous study [4]. Similarly, anti-MUSK autoantibodies of patients with myasthenia gravis that are also of the IgG4 subclass induce symptoms in passive transfer experiments of rats only after approximately 14 days of regular injection [20], whereas passive transfer experiments of IgG of patients with presumably acetylcholine receptor autoantibodies showed clinical symptoms after 2 to 7 days of injection and a reduction of amplitudes of miniature end-plate potentials as early as 12–21 h after a single injection [21], implying that acetylcholine receptor autoantibodies that mostly belong to the subclass IgG1 lead to more acute symptoms compared to anti-MUSK IgG4, probably due to an acute inflammatory response including complement deposition.

Conduction blocks as well as loss of F waves were not found in all animals injected with the same patient IgG in our study. This may be explained by technical reasons: intraneural injection might have only reached the epineurium in these animals or IgG might have not reached the whole cross-section area of the sciatic nerve, depending on the individual anatomy of each animal. Rare conduction blocks in control animals can be explained by iatrogenic lesions of the nerve by the injection itself, supporting the importance of a control group in this kind of experiments. Another limitation of our study is that total IgG instead of purified subclasses were used for injection. Therefore, it cannot be proven that higher effects in the acute patients are due to the IgG3 subclass. However, IgG3 was identified as the binding subclass in this patient, supporting the assumption that binding of IgG3 is pathogenic.

We did not find evidence of an inflammatory reaction induced by autoantibody binding, although complement deposition was detectable at the paranodes and is known to induce inflammatory response [22] and also did not find any relevant axonal degeneration. This finding argues against an inflammation-induced paranodal damage and leads to the suspicion of conduction block induced by autoantibody binding and temporary complement deposition only. The latter is supported by the quick recovery of the animals after a few days and the lack of spontaneous activity in EMG recordings.

## Conclusions

In summary, our data give evidence of an early pathogenic effect of anti-contactin-1 IgG3 rather than IgG4 after short-term exposure. Autoantibody binding and complement deposition rather than inflammatory responses or structural damage appear to underlie this effect. Different pathomechanisms may contribute to the clinical picture of anti-CNTN1-associated neuropathy, possible at different stages of disease and should be taken into account in therapeutic approaches.

## Additional files

Additional file 1: Video 1. Three-dimensional illustration of anti-Caspr staining of paranodes of an animal that was injected with IgG of the acute patient. (MP4 1332 kb)

Additional file 2: Video 2. Three-dimensional illustration of anti-Caspr staining of paranodes of a control animal. Paranodal volume is decreased in the animal that was injected with patient IgG (Additional file 1: video 1) compared to the control (Additional file 2: video 2). (MP4 1931 kb)

## Abbreviations

BSA: Bovine serum albumin
Caspr: Contactin-associated protein
CMAP: Compound muscle action potential
CNTN1: Contactin-1
ELISA: Enzyme-linked immunosorbent assay
NCS: Nerve conduction studies
NCV: Nerve conduction velocity
PBS: Phosphate-buffered saline
SNAP: Sensory nerve action potential
